# PEG Coated Fe_3_O_4_/RGO Nano-Cube-Like Structures for Cancer Therapy via Magnetic Hyperthermia

**DOI:** 10.3390/nano11092398

**Published:** 2021-09-15

**Authors:** Anoud Alkhayal, Arshia Fathima, Ali H. Alhasan, Edreese H. Alsharaeh

**Affiliations:** 1 College of Science and General Studies, Alfaisal University, P.O. Box 50927, Riyadh 11533, Saudi Arabia; aaalkhayal@alfaisal.edu (A.A.); arshiafathima92@gmail.com (A.F.); 2 National Center for Biotechnology, Life Science and Environment Research Institute, King Abdulaziz City for Science and Technology (KACST), P.O. Box 6086, Riyadh 11461, Saudi Arabia; aalhasan@kacst.edu.sa

**Keywords:** iron oxide nanoparticles, multi-functional nanocomposites, cubic nanoparticles, nano-magnetism, hyperthermia, breast cancer

## Abstract

Superparamagnetic iron oxide nanoparticles (SPIONs) have high saturation magnetization and are promising candidates for hyperthermia. They may act as magnetic heating agents when subjected to magnetic field in nano-based hyperthermia. In this work, cube-like Fe_3_O_4_ nanoparticles (labelled as cubic SPIONs) with reduced graphene oxide (RGO) nanocomposites were prepared by a microwave hydrothermal method. The shape and size of magnetic nanoparticles were controlled by varying synthesis parameters, including reaction time, pressure and microwave power. This study successfully synthesized cubic SPIONs nanocomposites with an average particle size between 24–34 nm. Poly-(ethylene) glycol (PEG) was used as a coating material on SPIONs to enhance biocompatibility. The RGO sheets provided a high surface area-to-volume ratio for SPIONs to be dispersed on their surface, and hence, they prevented aggregation of the SPIONs in the nanocomposites. Magnetically induced heating studies on the optimized nanocomposite (Fe_3_O_4_/RGO/PEG) demonstrated heating capabilities for magnetic hyperthermia application with a promising specific absorption rate (SAR) value of 58.33 W/g in acidic solution. Cytotoxicity tests were also performed to ensure low nanoparticle toxicity before incorporation into the human body. The results of the standard assay for the toxicity determination of the nanocomposites revealed over 70% cell survival after 48 h, suggesting the feasibility of using the synthesized nanocomposites for magnetic hyperthermia.

## 1. Introduction

Cancer is the uncontrolled growth of tissues and their rapid invasion without normal development and differentiation of cells [1]. Although it is necessary to minimize side effects and improve efficiency to devise a successful cancer treatment regime, it is also important to address the major limitations of several therapeutic agents such as poor solubility, rapid deactivation, unfavorable pharmacokinetics and limited biodistribution [1]. Nanomaterials have been widely studied for biomedical applications due to their high surface area-to-volume ratio, specific targeting capabilities and enhanced interaction with cells [1,2,3]. Nanomaterials have enhanced properties such as absorption, scattering, magnetic resonance or even their effective capability of delivering drugs, as compared to the bulk form of materials. Hence, various nanomaterials have also been tested for cancer treatment [1,4].

In contrast to chemotherapy and radiation, magnetic nanoparticles offer facile and effective cancer therapy treatment via hyperthermia with minimal side effects. Hyperthermia exposes malignant cells to heat and high temperatures (45–47 °C) as a means of weakening or destroying cancer cells [5]. As temperatures in this range cause functional and structural impairment to cancer cells, such as protein damage, it eventually triggers apoptosis in these cells [6]. Conventional hyperthermia employs an “outside-in” method of heating, where the majority of the heat is focused on the body’s surface, which then decreases in intensity as it moves away from the source [5]. The energy from the external radiation in conventional hyperthermia is dispersed and does not discriminate thermally between healthy or cancer tissues, and thus, it causes hot spot regions that may trigger a relapse of cancer [5,7]. Thermal discrimination is achieved when hyperthermia reaches only target malignant cells without causing any harm to healthy tissue [5]. The major advantage of (nano) magnetic hyperthermia is the thermal differentiation between healthy and cancer tissues. This thermal differentiation in tissues is attributed to the targeting capability of magnetic nanoparticles that could enter small areas and be guided via external magnetic fields [5]. Nanoparticles are used for targeted hyperthermia as they are able to reverse the direction of heat loss from outside-in to inside-out, making them the primary heating source in the modern hyperthermia method [5]. The two main heat generation mechanisms by magnetic nanoparticles are relaxation and hysteresis losses, which are crucial to understanding their effect on magnetic hyperthermia therapy. Magnetic hyperthermia therapy relies on the physical phenomenon of magnetic losses, which takes place when an alternating magnetic field (AMF) is applied to the sample. Magnetic and structural factors such as the particle size (d), saturation magnetization (M_s_), the viscosity of the sample (η) as well as the anisotropy constant (K_eff_) are the major parameters that influence magnetic losses [8].

Research on nanomaterials, specifically superparamagnetic iron oxide nanoparticles (SPIONs) such as Fe_3_O_4_ (magnetite) [9], has been extensively carried out for targeted drug delivery, medical imaging, cell targeting and hyperthermia therapy [3]. It was also clinically approved for human use [1,10]. SPIONs were chosen for their excellent biocompatibility and magnetic properties [1,4], with Fe_3_O_4_ nanoparticles favored for their high saturation magnetization [11], as the saturation magnetization was also proportional to the heating efficiency for magnetic hyperthermia [12]. The size of SPIONs is a crucial factor influencing its magnetic properties, with nanoparticles around 15–30 nm (average of 20 nm) required for superparamagnetism [12,13]. A significant challenge facing iron oxide magnetic nanoparticles is that the smaller the nanoparticles, the lower the saturation magnetization exhibited. SPIONs with high saturation magnetization capacities are desired in order to apply lower AMF during magnetic hyperthermia [12]. The morphological control of SPIONs, particularly nanocubes, allowed higher magnetization at small sizes than spherical shapes, even at low concentrations [12]. Other challenges for using SPIONs that need to be addressed include concerns over toxicological properties, long-term impact on human health [2] and the stability of the nanoparticle colloids due to their surface charge [9]. Although there are issues regarding the safety of SPIONs for clinical use, it is an interchange between toxicity and overall health benefits, and many factors contribute to this fact, as this study shows. The distribution of SPIONs is determined by charge, which also affects its internalization in the target cells [9]. In order to address these toxicity concerns and overcome the issue of aggregation, surface coating on the nanoparticles is employed.

Multifunctional properties were achieved using core-shell nanoparticles, where the shell (coating) could be used as a protective shield for the core or functionalized in a way that would reduce the cytotoxicity of the core [10]. Coating nanoparticles with biocompatible and biodegradable materials ensures the elimination of undesirable immune reactions that might be triggered by the toxicity of a material. Moreover, the best solution to bring the magnetic core-shell nanoparticles to a more isotropic state in a zero-magnetic field is to provide a coating around the particles [10]. SPIONs can be functionalized with many biocompatible polymers such as poly-(ethylene glycol) (PEG), dextran and polysaccharides to achieve better biocompatibility and stability in the blood [1]. Other nanomaterials such as graphene and its derivatives, including reduced graphene oxide (RGO), have been used with SPIONs as nanocomposites with enhanced hyperthermia efficiency [14]. The graphene family showed great potential in areas, such as gene delivery, loading anticancer drugs and antibacterial purposes, due to their enhanced surface properties and hydrophobic interactions [15]. The use of biocompatible 2D nanomaterials such as graphene to develop SPION nanocomposites is another strategy to overcome the challenges facing SPIONs and enhance their effectiveness. Multifunctional iron oxide nanomaterials using RGO have also been synthesized for application in photothermal therapy due to their high NIR absorbance [16]. The synergic effects of Fe_3_O_4_/RGO were demonstrated by the heating profiles of SPIONs/RGO nanocomposites for in vitro magnetic hyperthermia that showed 90% killing efficiency when exposed to an AMF. These nanocomposites also had enhanced drug release properties in acidic environments, as RGO was sensitive to pH due to residual carboxylic functional groups, which allowed for a pH-responsive release of chemotherapeutic drugs to the cancer cells. Thus, the nanocomposite had enhanced drug delivery properties, and hence, were potential candidates for therapeutic carriers and hyperthermia agents [17]. However, concerns over the toxicity of graphene and its derivatives have challenged the practical implementation of these nanocomposites. The graphene family, including graphene oxide (GO), is well known for unique interfaces with various functionalization sites that have enabled efficient drug delivery and diagnostics. Although the supposed cytotoxic effects of this family may lower its biocompatibility, it was found that the toxicity of GO depends on the concentration, the number of layers, density of oxygenation and cell-specific interaction [18]. Cell viability assays determined that pristine GO showed higher cytotoxicity effects than GO functionalized with Fe_3_O_4_ nanoparticles [19]. On the other hand, another factor that restrained toxicity was lower GO concentration, as studies seemed to agree that lower amounts of GO (<4 μg/mL) showed no toxicity [19]. Based on the literature, this work attempted to develop stable nanocube-like Fe_3_O_4_ NPs with graphene nanocomposites to capitalize on the synergistic effects between graphene and SPIONs for enhanced hyperthermia efficiency and possible drug delivery. The synthesized nanocomposites were then coated with PEG for enhanced biocompatibility. Moreover, the RGO sheets encapsulated the SPIONs as a means of protection from the acidic pH, while retaining their magnetization. The Fe_3_O_4_ nanocomposites were synthesized via a facile microwave hydrothermal synthesis for magnetic hyperthermia, with their sizes controlled within the required range for SPIONs as per literature (15–30 nm), by varying multiple synthesis parameters. The biocompatibility of the nanocomposites evaluated through cytotoxicity studies on human kidney cells and breast cancer cells showed cell viability of >70%, thus indicating its applicability for in vivo studies. The performance of these nanocomposites for application in magnetic hyperthermia was investigated in various dispersion media with promising SAR values of 58.33 W/g obtained in acidic pH solutions. 

## 2. Experimental Methods

The chemicals used in the synthesis of Fe_3_O_4_ were Iron(III) Chloride (FeCl_3_), reagent grade 97% (Sigma-Aldrich, St. Louis, MO, USA); Poly-(ethylene glycol) (PEG), average molecular weight 200 (Sigma-Aldrich, St. Louis, MO, USA); Hydrazine Hydrate (N_2_H_4_), 80% (Loba Chemie, Mumbai, India); and MilliQ H_2_O. RGO synthesis included the use of Graphite fine powder, extra pure (Merck, Darmstadt, Germany); Sulfuric acid (H_2_SO_4_), 95% (Sigma-Aldrich, St. Louis, MO, USA); Sodium Nitrate PRS (NaNO_3_) (Sigma-Aldrich, St. Louis, MO, USA); Potassium Permanganate (KMnO_4_), extra pure (Sigma-Aldrich, St. Louis, MO, USA); Hydrogen Peroxide (H_2_O_2_), 30% (Sigma-Aldrich, St. Louis, MO, USA); and Hydrochloric acid (HCl), 37% (Sigma-Aldrich, St. Louis, MO, USA). On the other hand, the dispersion medium used for hyperthermia tests includes Phosphate Buffered Saline (PBS) (Sigma-Aldrich, St. Louis, MO, USA); Buffer solution (acetic acid/sodium acetate), pH (20 °C) = 4.66 ± 0.01 (Merck, Darmstadt, Germany); and Dimethyl Sulphoxide (DMSO) (C_2_H_6_OS) (Loba Chemie, Mumbai, India). Chemicals were purchased from Sigma Aldrich (St. Louis, MO, USA), Merck (Darmstadt, Germany) and Loba Chemie (Mumbai, India) and were used without any purification.

The Fe_3_O_4_ graphene nanocomposites were prepared by microwave hydrothermal synthesis in CEM Mars 6 microwave (CEM, Charlotte, NC, USA) under varying conditions of concentration, pressure, temperature, power and time, as given below. 

### 2.1. Fe_3_O_4_ Nanoparticles (SPIONs) Synthesis

In a 25 mL beaker, 70 mM (90.8 mg) FeCl_3_ was mixed with 8 mL MilliQ water and magnetically stirred for 10 min at room temperature. Then, stirring was switched off, and 1 mL hydrazine hydrate was added. The samples were quickly transferred to CEM vessels and subjected to microwave irradiation under specific synthesis parameters: 900 W, 250 psi, 200 °C and 10 min. After the reaction, the samples were washed with deionized (DI) water in the centrifuge for five rounds under 3400 rpm, with 15 min per wash. The samples were then dried in the oven overnight at 80 °C.

### 2.2. Graphene Oxide (GO) Synthesis

The modified Hummer’s method was used to obtain graphene oxide (GO) [17]. Briefly, 125 mL of concentrated sulfuric acid was mixed with 2 g graphite at 0 °C. Then, 2.5 g of sodium nitrate was slowly mixed in, followed by the addition of 20 g of potassium permanganate while maintaining the temperature below 20 °C. The mixture was then heated to 35 °C for 2 h with vigorous stirring, followed by the addition of 230 mL of DI water with the temperature kept below 50 °C. The reaction was terminated with 20 mL of 30% hydrogen peroxide with subsequent color change to yellow. The product obtained was then washed with 100 mL of 10% hydrogen chloride solution, followed by extensive centrifuge washing with hot MilliQ water. The washing process continued until the pH of GO was near neutral. Sonication of the sample was performed for 10 min at a power of 6 in bath sonication (VWR^®^ Ultrasonic Cleaner, Avantor, Radnor, PA, USA). The sample was then dried at 60 °C overnight.

### 2.3. Fe_3_O_4_/RGO Nanocomposites Synthesis

The reduced graphene oxide (0.1% RGO) for the nanocomposites was derived from the addition of a homogenous aqueous GO solution (2 mg/mL) that was prepared by bath sonication to the mixture obtained in Fe_3_O_4_ synthesis. The initial procedure for nanocomposite synthesis was similar to that of Fe_3_O_4_ nanoparticles. After obtaining 70 mM solution of iron chloride, specific amounts of GO stock solution were added to obtain the desired weight percentage of Fe_3_O_4_ in the nanocomposite. The samples were sonicated for 60 min for complete exfoliation of GO and its interaction with the iron hydroxide flocs. Then, 1 mL hydrazine hydrate was added, and the samples were quickly transferred to CEM vessels and placed inside the CEM MW under optimized synthesis parameters: 900 W, 250 psi, 200 °C and 10 min. Samples were washed in the centrifuge as stated earlier, followed by oven drying at 80 °C overnight. For nanocomposites with PEG, an optimized amount of 8 mL PEG 200 was added after the GO solution during synthesis.

### 2.4. Characterization

The nanomaterials synthesized were characterized using X-ray diffraction (XRD) (Rigaku Miniflex 600, The Woodlands, TX, USA) and high-resolution transmission electron microscopy (HR-TEM JEM-2100F, JEOL, Boston, MA, USA). A copper grid was used for the HR-TEM sample preparation. The average particle sizes were estimated from the peaks obtained in the X-ray diffraction using the Scherrer equation. 

### 2.5. Procedures for Application Testing

#### 2.5.1. Cytotoxicity

Cytotoxicity testing in vitro for biocompatibility confirmation was conducted on two cell lines: Breast cancer cell line, MCF-7 (Michigan Cancer Foundation–7), and Embryonic kidney cell line, HEK-2 (Human Embryonic Kidney–293). The cells were purchased from (ATCC, Manassas, VA, USA). Cell culturing started with the preparation and aspiration of 1% penicillin/streptomycin (P/S) and 10% fetal bovine serum (FBS) media followed by its filtration for cell passaging with the addition of trypsin. The cell passaging process was initiated by aspirating the media from its original flask to a clean flask that was washed with PBS. It included the addition of trypsin, incubation, the addition of media, centrifugation, aspirating the supernatant and mixing the culture media along with the remaining pellet until homogeneous. After that, cell counting was conducted, where equal amounts of the cell suspension and Trypan blue were mixed together in order to dye the dead cells. The nanocomposites to be tested for cytotoxicity were suspended in media (10% FBS, 90% DMEM and 1% Penicillin) at a concentration of 200 µg/mL and was then added to the culture media prior to incubation for 24–48 h.

#### 2.5.2. Magnetic Hyperthermia

Easy Heat from Ambrell was used to test for magnetic hyperthermia. The SPIONs solution was placed in a glass tube at the center of a magnetic induction coil (8 turns, 3.7 cm diameter), held by a stand with a metal clamp having a plastic insulation cover to avoid any noise (heating effects) from the clamp itself. The trials were carried out by the application of the magnetic field in pulses with the field switched on for 5 min followed by the field switched off period for 10 s. The overall time of magnetic induction did not exceed 30 min per complete session. The obtained heating curves were fitted with a second degree polynomial model to estimate the initial slope (ΔT/Δt) used in the calculation of SAR value. For the SPIONs to be tested for hyperthermia therapy, a suitable medium must be used to provide an accurate simulation of the environment of cancer cells, preferably with minimal effects of particle aggregation. Thus, PBS (phosphate buffered saline), aqueous DMSO (dimethyl sulfoxide) solution (1:1) (DMSO:water) [20] and a pH buffer (pH = 4.66) were chosen as the media for the hyperthermia tests. Samples tested include Fe_3_O_4_/RGO and PEG-coated Fe_3_O_4_/RGO labelled Fe_3_O_4_/RGO/PEG. The dispersion media without any nanoparticles were tested as controls in this test.

## 3. Results and Discussion

### 3.1. XRD

**Figure 1:**
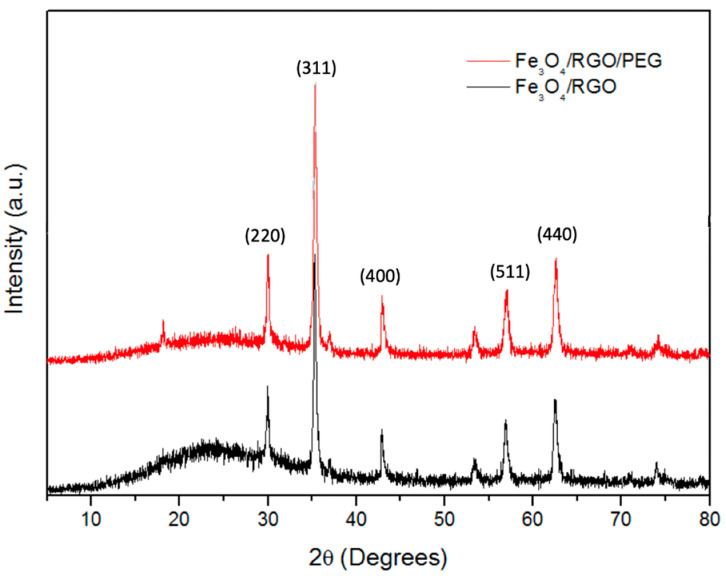
Comparative XRD analysis for Fe_3_O_4_ nanocomposites containing RGO and PEG.

The diffraction peaks and their positions of the following graphs of the SPIONs were consistent with the standard XRD data for magnetite syn(Fe_3_O_4_)-cubic-(ICSD 01-087-2334). These structures illustrated a cubic crystal system with potential cubic morphology. The planes for the diffraction peaks at 2θ of 30.11°, 35.47°, 43.14°, 53.43°, 57.05° and 62.61° were indexed as (220), (311), (400), (422), (511) and (440), respectively. Crystalline nanoparticles were prepared as observed from the sharp peaks in the XRD graphs (Figure 1). The indicator for the cubic shape was the relative intensity of the peaks at 30°/35°, where higher relative intensity is more likely to indicate cubic nanoparticles [11]. Considering the desired cubic shape, size and particle crystallinity, the optimal synthesis parameters were 900 W, 250 psi, 200 °C and 10 min of microwave irradiation. 

After coating with PEG, the average sizes of the SPIONs containing RGO and RGO/PEG are 34.8 ± 11.1 nm and 24.3 ± 4.1 nm, respectively. Hence, the optimal conditions chosen for SPIONs synthesis were also optimal for the Fe_3_O_4_/RGO/PEG samples, as observed from their size and high crystallinity (Figure 1). 

### 3.2. HR-TEM

**Figure 2:**
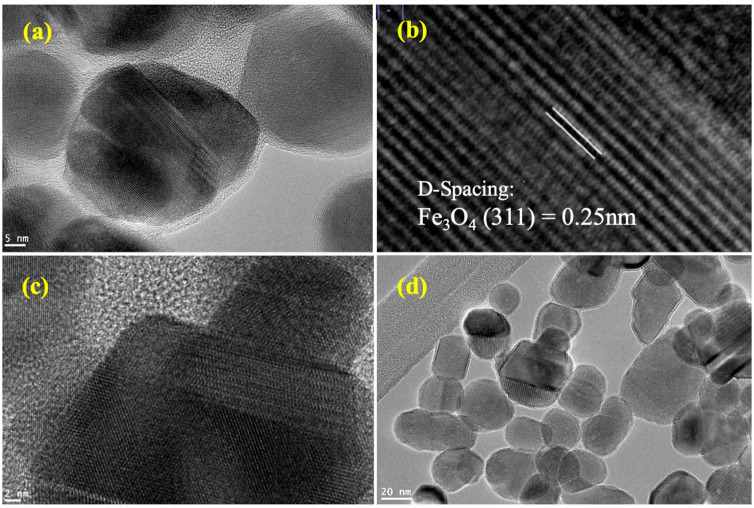
HR-TEM for (**a**) Fe_3_O_4_/RGO/PEG at the scale of 5 nm, (**b**) d-spacing of 0.25 nm for Fe_3_O_4_ nanoparticles, (**c**) Fe_3_O_4_/RGO/PEG at the scale of 2 nm showing clear cubic structure and (**d**) nanoparticles of the Fe_3_O_4_/RGO/PEG nanocomposite at the scale of 20 nm.

**Figure 3:**
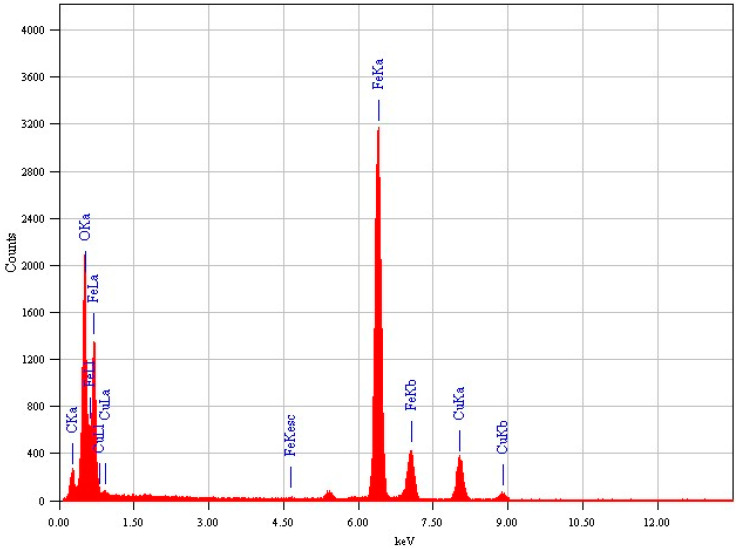
EDX spectrum of Fe_3_O_4_/RGO/PEG nanocomposite.

High-resolution transmission electron microscopy (HR-TEM) has become one of the leading tools for structural characterization of materials on the micro/nano levels [21]. In this research, TEM was used to investigate the crystallography of the synthesized SPIONs and their nanocomposites. A technique of phase-contrast imaging was used for high-resolution images of atom columns [21]. TEM also has a higher spatial resolution than scanning electron microscopy (SEM), and it confirmed the cube-like SPIONs, as observed from Figure 2. The cubic arrangement of the Fe_3_O_4_ atoms with a d-spacing of 0.25 nm was also observed, thereby confirming the cubic crystal system obtained from the XRD. PEG coating as a translucent outline surrounding the nanoparticles was also observed from these images. The synthesized nanocomposite was also detected with the elements Fe, O and C from EDX spectrum (Figure 3). The observed Cu peaks were attributed to the copper grid used for sample preparation.

### 3.3. Magnetic Hyperthermia

The favored heating agents in nano-magnetic hyperthermia are iron oxide nanoparticles due to a range of aspects such as their size-dependent magnetic properties, biocompatibility, ease of excretion, minimal toxicity and ease of functionalization [5]. Another advantage would be their movement inside the body in a non-invasive approach due to their intrinsic magnetic properties [22]. In magnetic nanoparticles, heat is generated by magnetization reversal caused due to an AMF along with the magnetic moment of the magnetic nanoparticles [13,23]. SPIONs exhibit Néel or Brown relaxation, where relaxation energetics and time for the reversal of magnetization parameters determined the relaxation method chosen by the SPIONs [5]. The Néel relaxation process occurred when nanoparticles were immobilized in the tumor. The characteristics of nanoparticles, such as particle size, coating layer and magnetic core, were generally optimized to maintain high SAR values in the presence of an AMF [5]. It was shown that 30 min of heating by magnetic nanoparticles (300 mg/mL) was sufficient in killing head and neck cancer cells in mice, with the additional advantage of no harm to healthy cells [24]. However, the necessity for high concentrations of magnetic nanoparticles to achieve sufficient heating effects in these studies was the major drawback for clinical use. 

**Figure 4:**
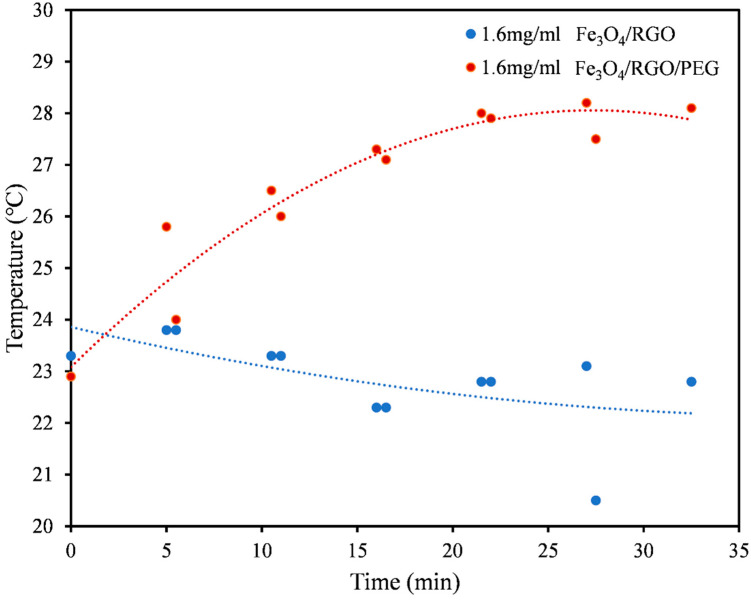
Heating profiles for 1.6 mg/mL solutions of Fe_3_O_4_/RGO (blue) and Fe_3_O_4_/RGO/PEG (red) in PBS medium at magnetic field of 200 A.

In this work, magnetic hyperthermia trials were conducted using the optimized SPIONs nanocomposites, while varying the currents (100 A, 150 A, 200 A) and in different media suspensions (PBS, aqueous DMSO, pH buffer) in order to test their heating efficiencies. Since the SPIONs will ultimately be tested with cultured cells, the safest media to test hyperthermia effects was PBS [25]. DMSO has been known as a well-tolerated pharmaceutical agent that manages pain caused by cancer. This factors in its biocompatibility as it contains chemical properties that help in its distribution in the body [20], and hence, it was chosen as one of the dispersion media for SPIONs nanocomposite suspensions to test for hyperthermia. Although DMSO concentration above 1% is toxic to cells, the magnetic hyperthermia tests were conducted in 50% aqueous DMSO solutions to investigate the maximum possible heating effects of the hydrophobic nanocomposites without any influence from particle aggregation. The pH buffer was used to simulate the acidic nature of cancer cells and their environment. In this study, the minimum concentration of SPIONs required for the magnetic heating effect was 1.6 mg/mL (Figure 4). For samples in PBS medium, it was observed that magnetic fields at 100 A and 150 A were insufficient in observing heating effects from the SPIONs. However, at the magnetic field at 200 A, the Fe_3_O_4_/RGO/PEG sample displayed good heating capacity, indicating a possibility to use it as a hyperthermia agent for cancer therapy (Figure 4). In contrast, the hydrophobic samples without PEG coating suffered from particle aggregation in PBS dispersion medium and hence, they did not exhibit any significant heating effects even at a magnetic field of 200 A. For further experiments with other media, 200 A was chosen as the optimal condition for magnetic field generation. The controls used were solvent medium without any nanoparticles in the solution. All the aqueous solvent controls will not show magnetically induced heating effects in the absence of SPIONs and hence, their heating curves will be the same as that of aqueous DMSO solution (Figure 4), where nearly constant temperatures at 20–25 °C were observed over the period of magnetic field exposure.

**Figure 5:**
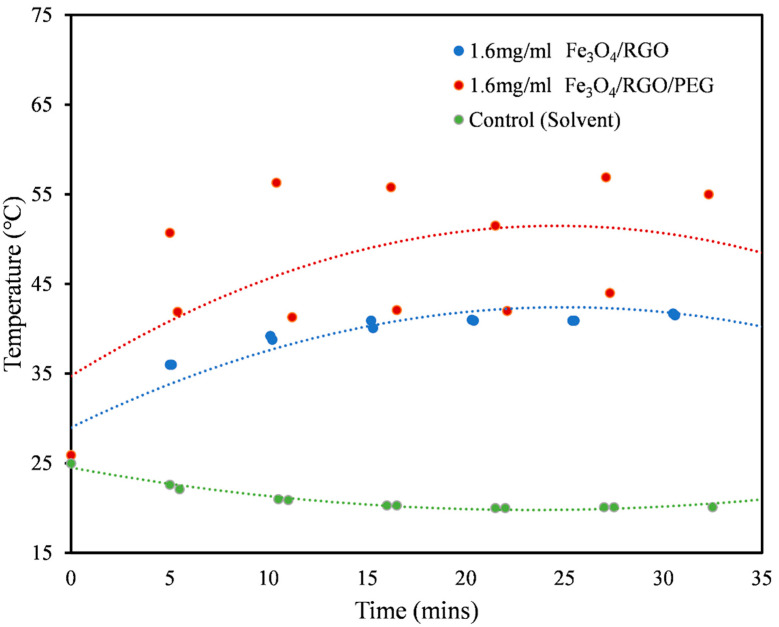
Heating profiles for 1.6 mg/mL solutions of Fe_3_O_4_/RGO (blue) and Fe_3_O_4_/RGO/PEG (red) along with the control (green) in aqueous DMSO medium at magnetic field of 200 A.

In contrast to the PBS medium, the Fe_3_O_4_/RGO nanocomposites in the aqueous DMSO medium showed an increase of 11 °C within the first 5 min of the magnetic field at 200 A, while the Fe_3_O_4_/RGO/PEG nanocomposites in the aqueous DMSO medium showed an increase of 24.8 °C in the same conditions (Figure 5). The aqueous DMSO medium was shown to be a better medium for investigating the heating effects of SPIONs nanocomposites due to its capability to disperse the hydrophobic SPIONs, given its amphipathic nature [20]. This medium would allow for enhanced heating effect via Néel and Brownian motion, as the particles were not restricted due to their aggregation. The Fe_3_O_4_/RGO/PEG nanocomposites in aqueous DMSO media had a larger increase in their temperatures due to minimal aggregation of the nanoparticles and increased dipole interactions that contributed to higher Néel-based heating effect [26].

**Figure 6:**
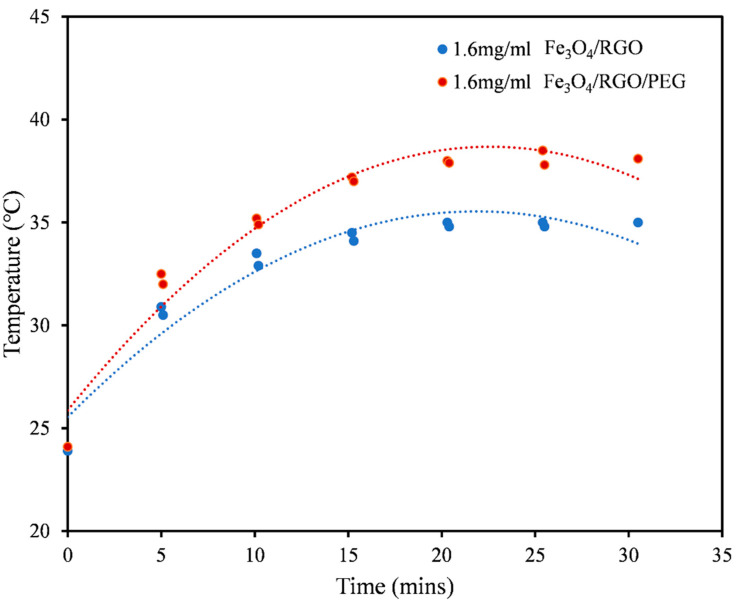
Heating profiles for 1.6 mg/mL Fe_3_O_4_/RGO (blue) and 1.6 mg/mL Fe_3_O_4_/RGO/PEG (red) in a 4.66 pH buffer at a magnetic field of 200 A.

In order to mimic the cancerous environment, pH buffer (4.66) was used as the dispersion medium for the magnetic hyperthermia test. The magnetically induced heating effect was not diminished in acidic media, although the heating effects of the nanocomposites in the pH buffer (4.66) was lower than that of the aqueous DMSO medium. However, the heating effects in the pH buffer were higher than those obtained in PBS medium due to lower nanoparticle aggregation in the organic acid buffer. Further investigation on the zeta potential of the nanocomposites will be performed to examine the effect of surface charges on nanoparticle aggregation. As seen with other suspensions, Fe_3_O_4_/RGO/PEG nanocomposites had better heating effects than nanocomposites without PEG (Figure 6), which shows promise in magnetic hyperthermia application for heating and terminating cancer cells. 

The PEG coating helped stabilize the particles and prevented aggregation of the nanoparticles that would lower the magnetic heating efficiency as observed in the nanocomposites without PEG coating. The morphology of SPIONs was a significant factor in the heat induction, with cubic nanoparticles having higher heat induction as compared to spherical shaped nanoparticles of similar sizes [20]. This increase in heat induction was due to the anisotropy of the particles, where the variation in its magnitude depended on the direction of measurement and their tendency to aggregate into a chain of cubic nanoparticles. This chaining effect was argued to display higher heating values in cubic nanoparticles than spherical nanoparticles [20], which was characterized by SAR values (also called Specific Loss Power—SLP). The SAR equation (Equation (1)), expressed in W/g^−1^, is a means of calculating the heat released by SPIONs under a magnetic field:

(1) $$\mathrm{SAR}\;=\;\frac{∆T}{∆t}\times \frac{C}{\mathrm{mFe}}$$

where C is the specific heat capacity of the dispersion, ∆T/∆t equals the initial slope of the time-dependent temperature curve (temperature increase per time unit) and mFe is the concentration of the colloidal nanoparticle suspension (g/L of iron).

Based on the results of the hyperthermia trials, the estimated SAR values of the overall experiments (Table 1) showed that Fe_3_O_4_/RGO/PEG had the highest SAR values in all dispersion media due to their smaller particle size affecting its magnetic properties and lower aggregation of the nanocomposites as compared to those without PEG coating. These SAR values were comparable to those observed in literature for the Fe_3_O_4_/RGO nanocomposites with spherical SPIONs (20 W/g at 335 Oe) [17]. It is noteworthy to mention the effect of the extrinsic parameters that influenced the heating results, besides the size and shape of the SPIONs, including the magnitude and frequency of the AMF as well as the viscosity of the dispersion. The ultimate goal of magnetic hyperthermia treatment is to reach the highest SAR values with the lowest number of nanoparticles and at the lowest possible frequency to not harm the patient undergoing the therapy [27]. Further investigation is required to gain insights into the effects of nanocomposite concentration, and particle aggregation on magnetically induced heating with experimental duplication is needed to validate the results.

### 3.4. Cytotoxicity

**Figure 7:**
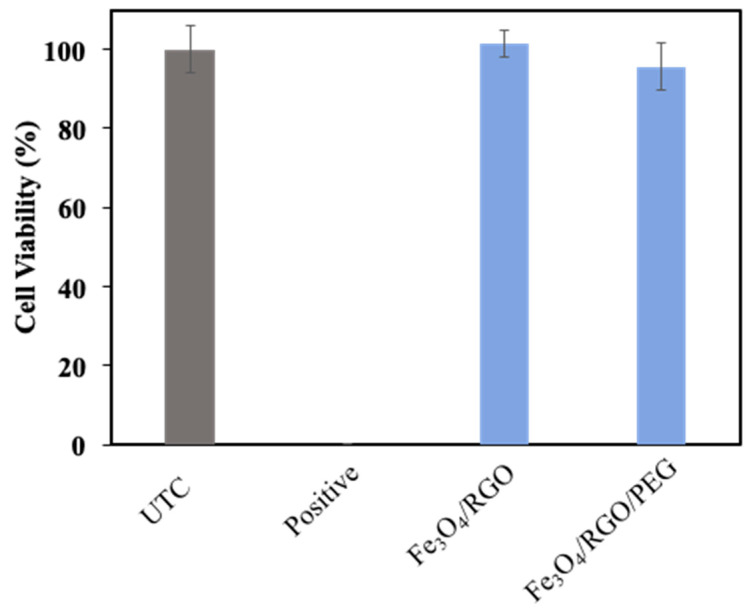
Cytotoxicity analysis of Fe_3_O_4_ nanocomposites in HEK-2 normal cells (24 h incubation).

**Figure 8:**
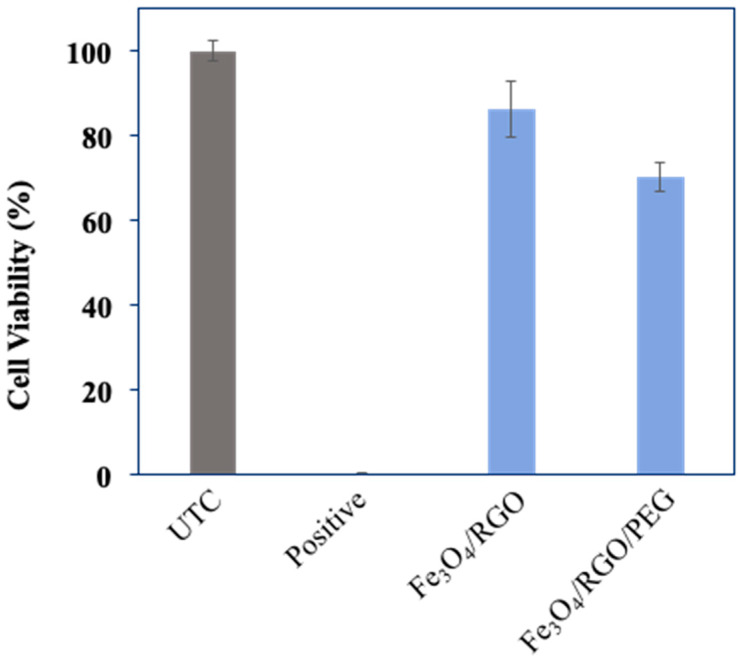
Cytotoxicity analysis of Fe_3_O_4_ nanocomposites in MCF-7 cells (24 h incubation).

Analyzing the cytotoxicity of the synthesized SPIONs was essential in order to eliminate the factors of harming the human body from the potential toxicity of SPIONs rather than curing it of cancer. Cytotoxicity tests were carried out on Breast cancer cell line MCF-7 (Michigan Cancer Foundation–7) and Embryonic kidney cell line HEK-2 (Human Embryonic Kidney–293) for Fe_3_O_4_/RGO and Fe_3_O_4_/RGO/PEG nanocomposites at a concentration of 200 µg/mL as a means of confirming the biocompatibility of the nanocomposites. The cell viability after 24 h incubation in HEK–2 cells (Figure 7) showed promising results, as all three nanocomposites did not show signs of inherent toxicity to the cancer cells, which was an indication that the SPIONs may not have toxic effects on healthy cells, as well. On the other hand, when MCF-7 cells were incubated with the SPIONs for 24 h (Figure 8), the cell viability was above 80% for Fe_3_O_4_/RGO, whereas Fe_3_O_4_/RGO/PEG had shown 70% viability. The MCF-7 cancer cells had lower cell viability in the presence of the SPION nanocomposites as compared to HEK-2 normal cells even though low concentration of the nanocomposites (200 µg/mL) was used in the cytotoxicity assay. This decrease in the viability of the MCF-7 cells may be due to cell interaction with the residual functional groups on RGO in the acidic cancer cell environment [17,18]. Further characterization is required to investigate the surface charge and pH-sensitive response of these nanocomposites to elucidate potential mechanisms of nanocomposite cytotoxicity in the cancer cells. 

Despite the known biocompatibility and non-toxicity of PEG, it was observed that PEG-based nanocomposites had lower viability as compared to the nanocomposites without PEG for all cytotoxicity studies. This decrease in cell viability may be due to the unreacted reagents adsorbed on the PEG-based nanocomposites during the synthesis process. It is anticipated that this issue may be addressed through more rigorous methods of preparation, such as dialysis-based washing to remove unreacted reagents from the synthesized nanocomposites.

**Figure 9:**
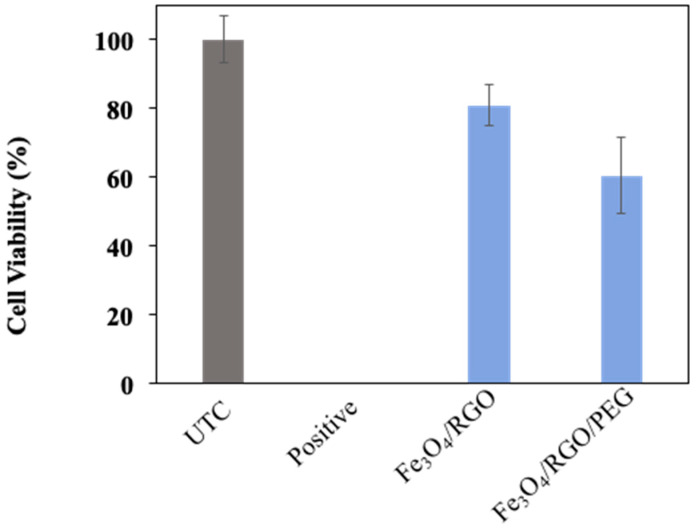
Cytotoxicity analysis of Fe_3_O_4_ nanocomposites in HEK-2 normal cells (48 h incubation).

Cell viability had decreased dramatically after 48 h of incubation (Figure 9), which indicated that the SPIONs may not be well tolerated for more than 24 h in the cancer cell environment and possibly the healthy cell environment, as well.

## 4. Conclusions and Future Outlook

The cube-like SPION nanocomposites synthesized in this work demonstrated their magnetic capacity for cancer therapy, where they could be used in magnetic hyperthermia to eliminate the tumor cells. The SPIONs were synthesized via a facile microwave hydrothermal method, which provided excellent reproducibility of results for producing larger quantities with desired sizes for superparamagnetism that had successful heating efficiencies similar to those found in the literature. SPIONs coated with RGO and PEG were synthesized and showed great promise in applications for in vivo testing and further cancer treatment modalities. The XRD analysis showed highly crystalline Fe_3_O_4_/RGO/PEG samples with cubic morphology and was corroborated by the HR-TEM images. As shown by magnetic hyperthermia results, Fe_3_O_4_/RGO/PEG performed better than Fe_3_O_4_/RGO due to lower nanoparticle aggregation in the dispersion media. Potential applications of near-infrared (NIR) laser were suggested instead of the typically operated AMF to clinically apply hyperthermia treatment, using the efficient photothermal effect of magnetic Fe_3_O_4_ nanoparticles [3]. This scheme was devised to overcome the demerits of the magnetic method that required the application of high current and voltage to achieve the desired heating effects. Future experiments will include dual-mode hyperthermia using magnetic field and NIR radiation with a focus on SPION/AuNP-based nanocomposites to exploit the potential plasmonic resonance in addition to the magnetic heating effects to realize the requisite temperatures for cancer hyperthermia treatment.

## Figures and Tables

**Table 1:** SAR values of Fe_3_O_4_/RGO nanocomposites in different media at 1.6 mg/mL concentration subjected to a magnetic field at 200 A.

Medium	Nanomaterial	SAR Values (W/g)
PBS (Figure 4)	Fe_3_O_4_/RGO	6.54
	Fe_3_O_4_/RGO/PEG	19.61
Aqueous DMSO (Figure 5)	Fe_3_O_4_/RGO	60.70
	Fe_3_O_4_/RGO/PEG	74.19
pH Buffer (Figure 6)	Fe_3_O_4_/RGO	45.76
	Fe_3_O_4_/RGO/PEG	58.33
